# Retraction Note: Extremely active Nano-formulation of Resveratrol (XAR™) attenuates and reverses chemotherapy-induced damage in mice ovaries and testes

**DOI:** 10.1186/s13048-023-01138-w

**Published:** 2023-03-24

**Authors:** Sagar Chhabria, Vaishnavi Takle, Nripen Sharma, Prashant Kharkar, Kshama Pansare, Anish Tripathi, Ashish Tripathi, Deepa Bhartiya

**Affiliations:** 1 Epigeneres Biotech Pvt. Ltd., Sun Mill Compound, Ikon House, B-Block, Senapati Bapat Marg, Lower Parel, Mumbai, Maharashtra 400013 India; 2 Department of Pharmaceutical Sciences and Technology, Institute of Chemical Technology, Matunga, Mumbai, 400 019 India; 3 Shobhaben Pratapbhai Patel School of Pharmacy & Technology Management, SVKM?s NMIMS, Vile Parle (West), Mumbai, 400 056 India

**Retraction Note: J Ovarian Res 15, 115 (2022)**

 10.1186/s13048-022-01043-8

The Editors-in-Chief have retracted this article at the corresponding author’s request. After publication, concerns were raised regarding high similarity between the images presented in Fig. 3B, D, E and F, and those previously published by different authors in Fig. 1C of [1]. The authors have been unable to locate the original images, and therefore no longer have confidence in the presented data.

Sagar Chhabria has not responded to any correspondence from the editor or publisher about this retraction. All other authors agree to this retraction.
